# Geographical distribution of at fault drivers involved in fatal traffic collisions in Tehran, Iran

**DOI:** 10.4178/epih.e2020002

**Published:** 2020-01-13

**Authors:** Amir Kavousi, Ali Moradi, Khaled Rahmani, Salahdin Zeini, Pegah Ameri

**Affiliations:** 1 Safety Promotion and Injury Prevention Research Center, School of Public Health and Safety, Shahid Beheshti University of Medical Sciences, Tehran, Iran; 2 Occupational Health and Safety Research Center, Hamadan University of Medical Sciences, Hamadan, Iran; 3 Liver and Digestive Research Center, Research Institute for Health Development, Kurdistan University of Medical Sciences, Sanandaj, Iran; 4 Accident Department, Traffic Police of Tehran, Tehran, Iran; 5 Deptartment of Epidemiology, School of Public Health, Hamadan University of Medical Sciences, Hamadan, Iran

**Keywords:** Spatial distribution, Traffic collisions, Drivers, Tehran

## Abstract

**OBJECTIVES:**

According to Traffic Police, about 35% of deaths and more than 50% of injuries caused by traffic collision in the roads of Tehran are among drivers and car occupants. This study was conducted to determine areas with the highest number of traffic collisions and perform spatial analysis of traffic collisions involving drivers in Tehran during April 2014 to March 2017.

**METHODS:**

The present study was a cross-sectional and descriptive-analytic research. In this study, all traffic collision that driver was accounted (100 percent or less) for crash occurrence which resulted in the death of at least one person (driver, pedestrian or passenger) were included in the analyses. Geographic information system software was used to show spatial distribution of events from zoning maps. Moran index was used in the mathematical analysis in order to determine the distribution pattern of the events from and Getis-Ord G statistics was applied to analyze the hot spots (high risk points).

**RESULTS:**

A total number of 519 traffic collisions were investigated in this study. Moreover, 283 cases (54.5%) of the incidents took place in direct routes and 236 cases (45.5%) occurred at intersections. The most frequent events were in the region 4 (57 cases) and the least frequent events were reported in the region 10 (6 cases). Moran statistics show that the distribution of the studied events significantly follows the cluster pattern (p<0.001).

**CONCLUSIONS:**

The northeastern and northwest margins of Tehran are the most prone areas for drivers involved with traffic collisions leading to death. Most traffic collisions leading to death take place at highways located at the entrance and exit points of Tehran and highways in regions 2 and 5.

## INTRODUCTION

Traffic collisions, as one of the main causes of death and disability in the world, cause annual deaths of 1.35 million people and injuries to tens of millions [1]. According to the study of the global burden of diseases, in terms of the number of years of life lost due to premature death or disability, the world’s leading road traffic injuries (RTI) were ranked ninth in 1991 and are projected to rise to third in 2020 [2]. Meanwhile, a significant proportion of the deaths and injuries caused by traffic collisions occur among drivers and car occupants, so that according to the World Health Organization (WHO) report in 2015, about 31% of deaths from traffic collisions occur for drivers and occupants of cars. The incidence of traffic collision involving occupants of cars in the European and Eastern Mediterranean countries is higher than in the rest of the world. In the European and Eastern Mediterranean countries, 51% and 45% of road traffic deaths occur in this category of road users [3].

According to the global study of the burden of diseases and injuries, in recent years, traffic collisions in the age group of 15 to 49 have been ranked first in terms of the number of years of life lost due to premature death in Iran [4]. The WHO’s 2013 report on road safety indicates that mortality rates from traffic collision in Iran are much higher than their global average (34.1 per 100,000 vs. 18.0 per 100,000) [1]. In Iran, in recent years, the use of cars for commuting within the cities has increased dramatically, and due to the social factors affecting traffic collisions, a significant proportion of RTI takes place especially among car drivers in the passages within the cities [5]. According to Traffic Police statistics, about 30% of deaths and more than 50% of RTI in the major road to Tehran have taken place among drivers and car occupants, and more than 35% of the deaths from such incidents have been associated with motorcyclists [6]. Despite the high percentage of road traffic crashes, there has been no significant analysis of traffic collisions leading to deaths in this group, and there is no evidence of spatial distribution of traffic collisions leading to deaths drivers in Iran. Accordingly, the aim of the present study was to perform spatial analysis of traffic collisions and determine the prone areas of traffic collisions involving drivers in Tehran during the period of April 2014 to March 2017.

### Studies performed in local context

Moradi et al. [6] indicated that there had been a cluster distribution of events in the areas under study. Soltani & Askari [7] investigated spatial autocorrelation of traffic collisions in Shiraz City in south of Iran using geographic information system (GIS), Moran statistics, as well as Getis-Ord Gi^*^. The results of their study indicated that there was a cluster spatial and temporal distribution of traffic collisions during the years 2009 to 2015 in traffic areas of Shiraz City. Shafabakhsh et al. [8] investigated the pattern of events distribution within the city of Mashhad using kernel density estimation (KDE). Their study reflected the cluster pattern of events distribution.

Aghajani et al. [9] studied spatial and temporal patterns of traffic incidences in Ilam Province located in west of Iran. Their study indicated the cluster distribution of events. Soori & Moradi [10] performed in 2016, the study showed that the severity of traffic collisions is higher in freeways and highways, non-residential areas, during the sunrise, vertical and horizontal curves, outside the pathway and in unstable weather. Ayati & Abbasi [11] used GIS in order to investigate traffic collisions in highways within Mashhad City during the years 2006 and 2007.

### Location of the study

Tehran is ranked 25th among the most populated cities of the world and according to statistics, its development and growth will increase until the year 2030 [12]. Tehran is the largest city and capital of Iran, located in the northern part of the country on the southern slope of the Alborz Mountains. Tehran is geographically located at 51 degrees 17 minutes to 51 degrees 33 minutes east and 35 degrees 36 minutes to 35 degrees and 44 minutes’ north latitude. The area of Tehran is more than 612 km^2^. In Tajrish square, the height is about 1,300 m and in railroad square, it is 1,100 m above the sea. This difference in the level is due to the size and breadth of the city. Based on the first official census that took place in 1956, Tehran, with a population of 1,560,934, was the most populous city in Iran [13]. Based on the results of the general census of population and housing in 2006, Tehran’s population was 7,803,883 [14]. The current population of Tehran is estimated to be 8,089,766 people. This population consists of only the residents and several million people are added to this fixed population during the day, particularly in working hours. The administrative structure of Iran is concentrated in Tehran. Tehran is divided into 22 regions, 134 districts including Ray and Tajrish, 370 neighborhoods, and 560 traffic regions [15].

## MATERIALS AND METHODS

The present study was a cross-sectional and descriptive-analytic research. The data necessary for conducting the study were extracted from the databases of the Traffic Police of Tehran. In this research, all traffic collisions that at fault involved driver (100% or less) for traffic collisions occurrence and also the traffic collisions resulted in the death of at least one person; driver, pedestrian or passenger (at fault drivers involved in fatal traffic collisions) were included in the analysis. It should be noted that, traffic collisions which occurred in the 22 districts of Tehran were entered to the study.

### Statistical analysis

This study was carried out in two sections of descriptive and analytic, using SPSS version 20 (IBM Corp., Armonk, NY, USA) and ArcGIS version 10.2 (ESRI Inc., Redlands, CA, USA) software. To describe the quantitative data, absolute and relative frequencies were used and to show distribution of events based on spatial complications, zoning maps were applied. Mathematical analyses were done in the following order:

(1) Determining the location of the events: The exact address of the location of at fault drivers involved in fatal traffic collisions was extracted using their sketch maps at the traffic police database.

(2) Preparing geographic data file: Depending on the location of the traffic collisions, the geographic coordinates of their location were recorded according to the Universal Transverse Mercator coordinates system and were then entered into the computer.

(3) Preparing zoning maps: Using Arc-Map software, different layers of geographic information were combined and the zoning maps were extracted.

(4) Determining distribution pattern of events: Using the Moran’s I-index, the distribution pattern of traffic collisions was investigated and analyzed regarding being in a cluster or distributed pattern. Moran I-index is the most commonly used index for measuring spatial autocorrelation between charges of land, phenomena, and events. Moran I-index examines the pattern of distribution of these charges by considering the attribute values studied in terms of cluster or distributed patterns. Moran I-index is calculated through the following relationship:

$$I=\frac{N\sum_{i=1}^{n}\sum_{j=1}^{n}w_{\mathrm{ij}}\left(x_{i}-\bar{x}\right)\left(x_{j}-\bar{x}\right)}{\left(\sum_{i=1}^{n}\overset{n}{\underset{j=1}{\sum w_{\mathrm{ij}}}}\right)\sum_{i=1}^{n}{\left(x_{i}-\bar{x}\right)}^{2}}$$

Where, *N* is the number of observations (points or polygons), X the mean of the variables, X_i_ the variable size at a given location, X_j_ the variable size at another location, and W_ij_ is the weighting index of the location I relative to the location of j. Based on the distribution of normal frequency with the following equation:

$$Z=\frac{I-E\left(I\right)}{Serror\left(I\right)}$$

In which *I* is the size of the Moran statistics calculated from the sample, *E(I)* the expected value of *I*, assuming it is random, and *S_I_* the standard error, and the *Z* size of this statistic is calculated. Accordingly, a statistical test can be also done. In this test, the zero assumption includes the absence of autocorrelation and the assumption of the existence of spatial correlation. If the calculated Z size is greater than 1.96 or less than -1.96, the zero assumption is rejected with 95% confidence.

The size of the Moran statistics varies between -1 and +1. The value of +1 represents a completely single-polar (cluster) pattern in which the zero value indicates the random or multi-polar aggregate pattern, and the value of -1 denotes the distributed pattern. The higher the coefficient, the higher the accumulation, while the lower the number, it indicates the distribution [16].

(5) Analysis of hot spots: In order to perform these analyses, Getis-Ord Gi^*^ statistics were calculated for the traffic collisions under study. The relevant maps were also extracted. The Z-size of this statistic indicates how the variable studied is spatially distributed in cluster form, so that its cluster status could be statistically significant. The Getis-Ord Gi^*^ statistic is calculated by the following equation:

Gi*=∑j=1nwi,jxj-X∑wi,jj=1nSn∑wi,j2j=1n-∑wi,jj=1n2n-1

Where *X_j_* is the number of the events for the geographic unit *j*, *W_ji_* the spatial weight between the geographic units i and j, n the number of geographic units, X and *S* are the average and standard deviations of the variable, respectively, and are calculated based on the following relationships:

X ¯= ∑xjj=1nnS = ∑xj2j=1nn -X2

In the present study, this statistic has been used to indicate areas that are at the high, low, or average level of the total community in terms of drivers-related traffic collisions. The interpretation of the G-values was based on the comparison of observed and expected values. If the observed value is greater than the expected value in a region, that region is included in the hot or risky points, and if the observed value is less than the expected value in a region, that area is considered as a cold or not prone area [17].

### Ethics statement

The design of the study was evaluated and confirmed by Ethic Committee in Shahid Beheshti University of Medical University (IR.SBMU.REC.1396.85).

## RESULTS

**Figure 1.**
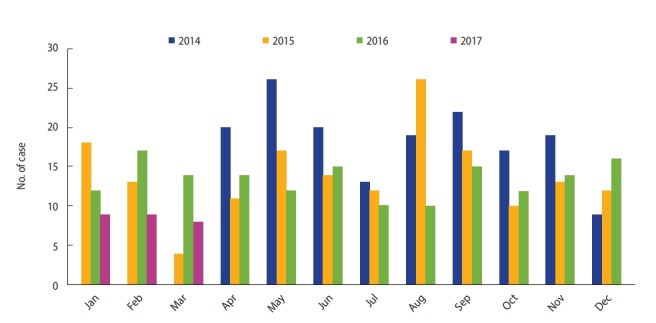
Distribution of fatal traffic collisions associated with drivers in Tehran, April 2014 to March 2017.

In this study, a total number of 519 events were investigated in Tehran during the period of April 2014 to March 2017. The number of traffic collisions has decreased from April 2014 to March 2017, with the most and the least at fault drivers involved in fatal traffic collisions for 2014 (200 traffic collisions) and 2016 (144 traffic collisions), respectively. The most frequent events were in May and August (55 cases) and the lowest frequency was in March (26 cases). Figure 1 indicates the trend of at fault drivers involved in fatal traffic collisions in separate months during the period of April 2014 to March 2017 in Tehran.

**Figure 2.**
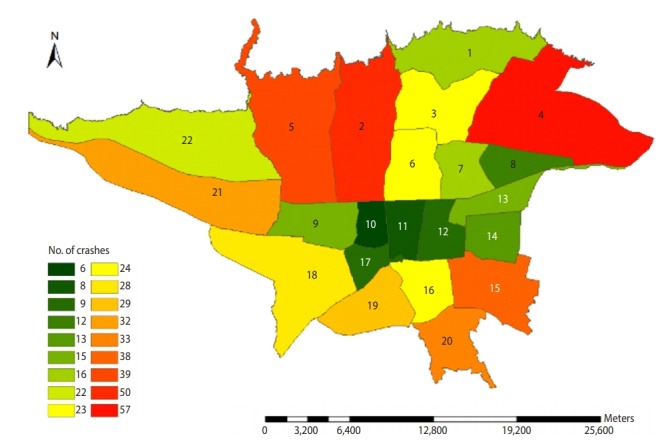
Distribution of studied traffic collisions in Tehran based on separate urban areas during April 2014 to March 2017.

The most frequent events were in the region 4 (57 cases) and the least frequent incidents were in the region 10 (6 cases). Figure 2 shows the distribution of the events studied in Tehran in April 2014 to March 2017 across the 22 regions.

**Figure 3.**
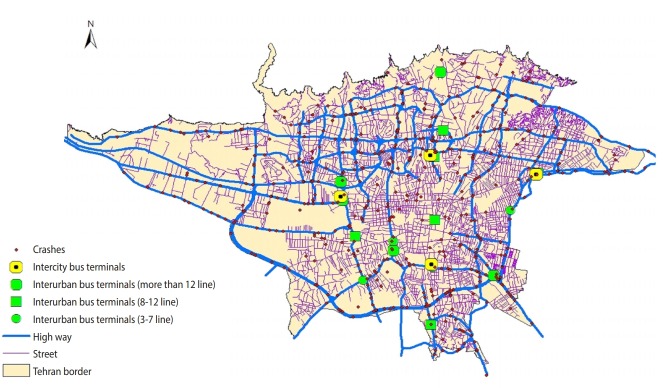
Distribution of studied traffic collisions in Tehran and their location relative to highways and bus terminals during April 2014 to March 2017.

In this regard, 283 cases (54.5%) of the traffic collisions had occurred in direct routes and 236 cases (45.5%) had happened at intersections. Moreover, 407 cases (78.4%) of the traffic collisions had occurred on highways. Figure 3 shows the distribution of the studied traffic collisions in the city of Tehran and their position relative to highways and bus terminals between and within cities. This figure indicates that there is a high density of traffic collisions around the bus terminals. We estimated the correlation between length of highways located in each of the regions and number of traffic collisions that occurred (r= 0.81, p< 0.001).

**Figure 4.**
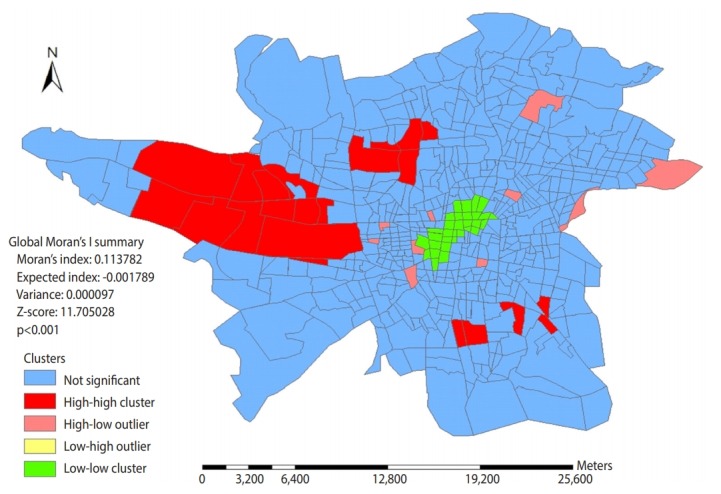
Distribution pattern of fatal traffic collisions in Tehran based on traffic areas during April 2014 to March 2017.

The traffic areas 335,484,481, and 202 have the highest frequency of at fault drivers involved in fatal traffic collisions during the study period. Figure 4 shows the distribution pattern of fatal traffic collisions related to drivers in traffic areas. The size of the Moran statistic indicates that the distribution of the studied events significantly follows the cluster pattern (p< 0.001).

**Figure 5.**
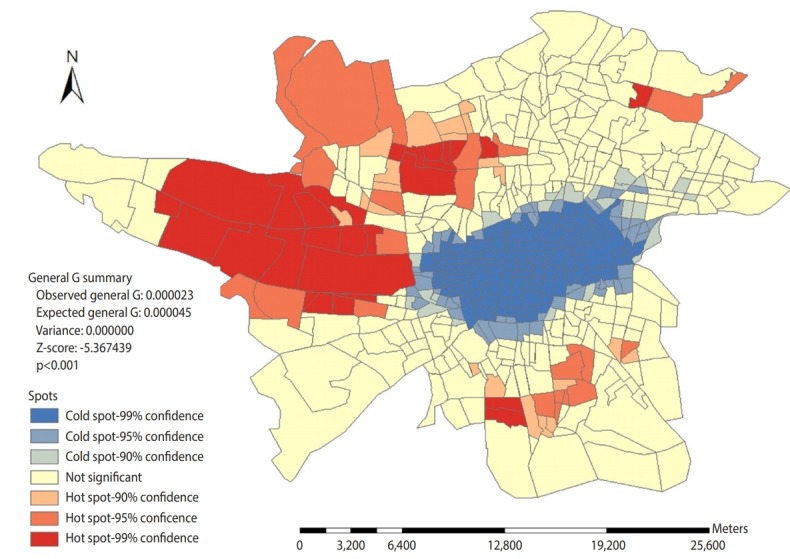
Spatial distribution of low and high spots regarding density of fatal traffic collisions based on traffic areas in Tehran during April 2014 to March 2017.

Figure 5 shows that the prone areas in Tehran regarding fatal RTI are located in the western, southern, northern, and almost eastern suburbs of the city, while the not prone areas are concentrated in downtown of Tehran. Getis-Ord General G statistics indicate that the distribution of high risk and not prone areas is statistically significant (p< 0.001).

## DISCUSSION

The present study which aimed to investigate the spatial analysis of at fault drivers involved in fatal traffic collisions in Tehran has been the first study which is specifically focused on deaths caused by traffic collisions in Tehran during different months from April 2014 to March 2017 with no definite trend.

In 2014-2015, the highest frequency of cases has been were in May, and the lowest in March, while during 2016-2015, the highest frequency has been reported in August and the lowest in October, and finally, during 2016-2017, June and September have had the highest and March the lowest number of events.

Nevertheless, in the period of April 2014 to March 2017, the number of incidents in summer, especially in the months of August and September, was much higher than in winter, especially in March. Some studies have shown higher pedestrian volume, increasing of the number of pedestrians in a street, increases the frequency of traffic collisions significantly [18-20]. On the other hand, with the increase in traffic volume, the frequency of traffic collisions, including at fault motorcyclists involved in traffic collisions, is significantly increased [21,22]. The reason for these seasonal differences can be the changes in pedestrian and motorcyclist volumes. Therefore, one of the ways to reduce deaths due to at fault drivers involved in fatal traffic collisions in Tehran is to better manage pedestrian and motorcyclists commute.

This study showed that about 80% of the traffic collisions have occurred on highways and freeways. Highways located at the main entrance and exit of the west and south of Tehran, particularly Azadegan highway and Karaj Special Road account for more traffic collisions. These findings are consistent with the results of Wier et al. [23] in San Francisco and Mueller et al. [24] in Washington, which indicated that the number of traffic collisions is higher on highways and junctions with heavy traffic. Considering that the traffic load of highways located in the west and south of Tehran is more than in other regions and in some of these passages there is a sharp mixing of different vehicles, one of the reasons for the high frequency of traffic collisions in these areas can be higher traffic load and a mix of different vehicles. Therefore, it is critical to determine the reasons for this issue by conducting further studies and to take necessary measures regarding the organization of the transport of vehicles, including the separation of light and heavy vehicles in the highways of the west and south of Tehran.

The distribution of the events surveyed in map 1 relative to the location of the terminals between and within cities indicates that the density of traffic collisions is relatively higher at the end of the passageways to Azadi terminal, particularly the Special Road of Karaj, the Afsariyeh Bridge, and the beginning of Imam Reza Highway. Therefore, one of the reasons for the high incidence of deaths in the south and west of Tehran can be associated with the presence of inter-city terminals in west (Azadi) as well as within-city terminals of Afsariyeh in these areas. Moreover, distribution of traffic collisions is also high compared to other within-city passenger terminals, especially for those in the vicinity of interurban terminals. Thus, passages around within-city terminals are considered to have a higher risk regarding incidents leading to deaths of drivers.

The comparison of the 22 regions of Tehran in terms of the frequency of traffic collisions leading to deaths indicates that the highest frequency of these incidents has occurred in regions 2 and 4 (107 cases), which accounts for 20.6% of all cases. Given the fact that most traffic collisions in regions 2 and 4 highways have occurred in highways, one of the reasons for more traffic collisions involving drivers in regions 2 and 4 could be the relative highway lengths. The highways of these areas with a length of about 105 km cover about 20% of the highways of Tehran [15].

The results indicated that high risk traffic areas regarding fatal traffic collisions were also located in the west and south of Tehran during the study years. The high Moran statistics indicate that distribution of traffic collisions has been based on the cluster pattern of 560 Tehran traffic areas. In other words, the location of the incidents in Tehran has not been random. Furthermore, Figure 5 shows that high risk areas are located in western and southern parts of Tehran, and not prone areas are concentrated in the center of Tehran. Given that according to Getis-Ord General G statistics, the distribution of high risk and not prone areas is also statistically significant, it can be said that certain factors contribute in the non-random distribution of traffic collisions resulting in deaths in Tehran as well as the high incidence of such events in prone areas among which environmental factors can play a significant role given the environmental and structural differences between the prone areas and low-risk areas that are located in the center of Tehran.

Similar studies [25-30] in other parts of the world have all shown that environmental factors can play a major role in the non-random distribution of traffic collisions.

### Strengths and weaknesses of the study

This study is one of the few studies in Iran that has used sketch maps depicted by police experts to conduct an applied research to determine the prone areas in a metropolis. In this study, with the help of traffic collisions, the geographic coordinates of their location were accurately recorded and consequently, preparation of zoning maps was possible in order to determine the distribution of at fault drivers involved in fatal traffic collisions and their location relative to other spatial complications.

This study was conducted only using data from traffic collisions recorded by the traffic police of Tehran. Comparison of police and forensic statistics shows that the actual number of deaths in Tehran has been higher than that of traffic police data, and the information associated with a number of traffic collisions to deaths is lacking in police databases [6]; therefore, access to spatial information associated with the location of the events is not possible through police sketch maps.

In this type of study, it is better to determine the relevance of the deaths of the injured as a result of traffic collisions based on the time of death after the incident, which varies from 6 days to 1 month in different countries. In Iran, this interval is defined as one month. However, due to the lack of consistency between police and forensic data in this regard, the relevance of the deaths of injured people as a result of traffic collisions was determined by traffic collisions based on traffic police data.

In conclusion, west, south, northwest and east of Tehran are the most prone areas in terms of traffic collisions leading to deaths of drivers. Most traffic collisions resulting in deaths in Tehran take place on highways, especially the highways located at the main entrance and exit points of Tehran, namely, 4, 15, 20, and 21 urban areas and highways of regions 2, 4, and 5. Therefore, the highways of Tehran are the most prone passages for traffic collisions. The cluster distribution of traffic collisions in terms of 560 traffic areas in Tehran and the presence of prone areas in terms of the events studied in the west and south of Tehran indicate significant environmental factors in the incidence of traffic collisions leading to death. As a result, further studies are required to identify these factors and organize the transport of vehicles in this areas.
